# How to Differentiate General Toxicity-Related Endocrine Effects from Endocrine Disruption: Systematic Review of Carbon Disulfide Data

**DOI:** 10.3390/ijms23063153

**Published:** 2022-03-15

**Authors:** Nathalie Printemps, Brigitte Le Magueresse-Battistoni, Sakina Mhaouty-Kodja, Catherine Viguié, Cécile Michel

**Affiliations:** 1 Risk Assessment Department, ANSES, 14 Rue Pierre et Marie Curie, 94701 Maisons-Alfort, France; cecile.michel@anses.fr; 2 Univ-Lyon, CarMeN Laboratory, INSERM U1060, INRAé U1397, Université Claude Bernard Lyon 1, 69310 Pierre-Bénite, France; brigitte.lemagueresse@inserm.fr; 3 Sorbonne Université—CNRS, INSERM, Neuroscience Paris Seine—Institut de Biologie Paris Seine, 75005 Paris, France; sakina.mhaouty-kodja@sorbonne-universite.fr; 4 Toxalim (Research Centre in Food Toxicology), Université de Toulouse, INRAE, ENVT, INP-Purpan, UPS, 31027 Toulouse, France; catherine.viguie@inrae.fr

**Keywords:** endocrine disruptors, carbon disulfide, thyroid disruption, neurotoxicity, cardiotoxicity

## Abstract

This review provides an overview of the assessment of the endocrine disrupting (ED) properties of carbon disulfide (CS_2_), following the methodology used at the European level to identify endocrine disruptors. Relevant in vitro, in vivo studies and human data are analyzed. The assessment presented here focuses on one endocrine activity, i.e., thyroid disruption, and two main adverse effects, neurotoxicity and cardiotoxicity. The data available on the different ED or non-ED modes of action (MoA), known to trigger these adverse effects, are described and the strength of evidence of the different MoA is weighted. We conclude that the adverse effects could be due to systemic toxicity rather than endocrine-mediated toxicity. This assessment illustrates the scientific and regulatory challenges in differentiating a specific endocrine disruption from an indirect endocrine effect resulting from a non-ED mediated systemic toxicity. This issue of evaluating the ED properties of highly toxic and reactive substances has been insufficiently developed by European guidance so far and needs to be further addressed. Finally, this example also raises questions about the capacity of the technics available in toxicology to address such a complex issue with certainty.

## 1. Introduction

There is a real societal interest in knowing which substances should be identified as an endocrine disruptor (ED). The available lists for potential endocrine disruptors, such as the one reported by the French Agency Environmental and Occupational Health and Safety (ANSES) [1], include many substances of concern for their potential ED properties. Some of these substances are already classified for severe health hazards related to toxicities other than endocrine disruption. In the European Union and based on the World Health Organization, a substance should be considered as an ED if it fulfills three criteria. First, it shows an adverse effect in an intact organism or its progeny/ in non-target organisms. The adverse effect is defined as a change in the morphology, physiology, growth, development, reproduction or life span of an organism, system or (sub)population that results in an impairment of functional capacity, an impairment of the capacity to compensate for additional stress or an increase in susceptibility to other influences. Second, it has an ED mode of action (MoA): i.e., it alters the function(s) of the endocrine system. Third, the adverse effect is a consequence of the ED MoA [2].

Beyond the debate on the benefit in terms of public health of spending scientific resources to perform ED assessment in addition to other hazard identification, several challenges have to be faced to identify endocrine disruptors. The first challenge is related to data availability. Indeed, most of the regulatory toxicology tests are performed at doses high enough to produce general toxicity while EDs can act at environmental low doses, especially during the most sensitive periods of life [3]. A second major challenge lies in the strict dependence of the adverse effect on an ED MoA [4]. Whether the adverse effect is related to an ED mechanism or may be the result of non-endocrine mechanism is defined as specificity in the European guidelines [5]. This raises a critical issue on how to categorize a substance for which systemic toxicity is observed jointly to endocrine alterations. Indeed, it may be complex to distinguish between a specific ED effect and a non-specific secondary ED effect resulting from systemic toxicity. This is all the more critical since the available data do not always allow one to describe with certainty the chronology of endocrine-mediated events compared to other non-ED events.

The evaluation of carbon disulfide (CS_2_) as a potential endocrine disruptor perfectly illustrates these challenges. CS_2_ (CAS No. 75-15-0) is an industrial chemical mainly used in the manufacture of regenerated cellulose and viscose or as an intermediate in the manufacture of other chemicals such as dithiocarbamate pesticides or carbon tetrachloride. The annual production in Europe is above 100,000 tons per year [6]. The substance is a highly volatile liquid at ambient temperature. Therefore, workers are expected to be occupationally exposed to the substance either by inhalation or through the dermal route. In addition, a human may also be exposed at low doses via the environment (air, water) near the manufacturing facilities.

According to the current European Union harmonized classification and labelling regulation [7], and the lead-registrant self-classification [6], the substance is classified as suspected for reproductive toxicity (damaging fertility and unborn child development). CS_2_ is also classified for its severe health effects on the nervous system, the cardiovascular system and the eyes after prolonged or repeated exposure.

The current occupational exposure limit was set at 15 mg/m^3^ [8]. The limit was based on the most sensitive effects in human, i.e., neurotoxicity and cardiotoxicity [9]. In addition, epidemiological studies on occupational cohorts exposed to CS_2_ also reported effects of concern (i.e., at low dose) on reproductive function [10,11,12,13,14,15,16,17,18,19] or thyroxine (T4) levels [20,21,22,23]. CS_2_ has been identified as a potential ED in different lists, such as the “Database of endocrine disrupting chemicals and their toxicity profiles” list (DEDuCT) [24] or “The Endocrine Disruption Exchange” list (TEDX) [25].

Our goal was to evaluate the ED properties of CS_2_ to help decide if extra risk management measures should be put in place to ensure safe use of this chemical. The assessment was based on the EU criteria and the method proposed by the joint European Food safety Authority/European chemical Agency (EFSA/ECHA) guidance document [5]. We have identified the thyroid function as one of the most likely targets of CS_2_-induced endocrine disruption. We aimed to establish the biological plausibility of the link between thyroid function disruption and the most critical adverse effects of CS_2_ and to discriminate it from a secondary non-specific consequence of other toxic mechanism.

Our ultimate goal is to contribute to build a roadmap allowing one to discriminate between endocrine disruption and endocrine alterations resulting from non-specific toxicities.

## 2. Methods

Although CS_2_ is an industrial chemical, assessed under the Regulation (EC) No. 1907/2006 concerning the registration, evaluation, authorization and restriction of chemicals (REACh), the method used for identification of its ED properties was adapted from the EFSA/ECHA guideline document developed under the pesticide and biocide regulations [5]. The strategy is based on the three conditions provided in the ED criteria: adversity, endocrine activity and a biological ED MoA as a link between the adverse effect and the endocrine activity. In line with this guideline, La Merill et al. (2020) proposed a method focusing on key characteristics for the identification of ED properties rather than specific MoA. These features allow for a broad and holistic review of the mechanistic evidence [26].

Information was gathered based on the data available in the REACh registration dossier for CS_2_. In addition, a literature search was performed on CS_2_ in Pubmed^®^ using the search term “75-15-0 [CAS] in abstract-title and key words” in papers from 2013 to 2019. A set of inclusion and exclusion criteria was then applied to select the relevant studies (Table S1 of supplementary material). Previously published international evaluations were also taken into consideration [9,27,28,29,30,31]. A search has also been conducted in the U.S. Environmental Protection Agency’s Endocrine Disruptor Screening Program [32] to determine the potential for CS_2_ to interact with estrogen, androgen or thyroid (E, A or T) bioactivity based on in vitro high-throughput screening assays.

**Figure 1:**
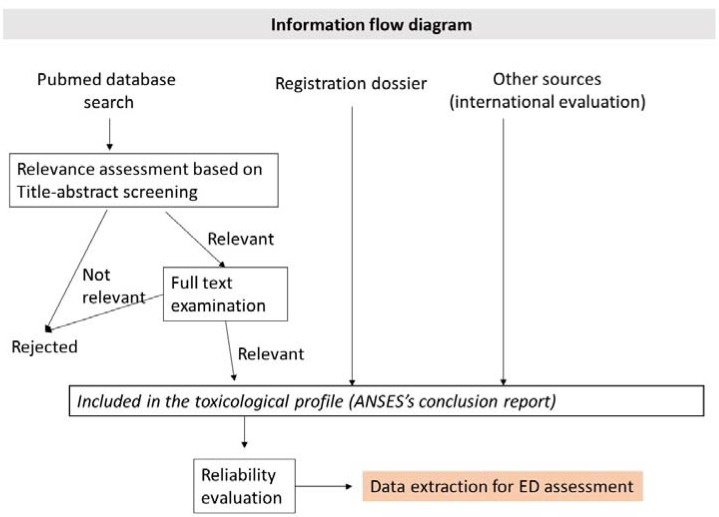
Information flow diagram.

In vitro, in vivo animal data, mechanistic data and human data relevant for the assessment of ED properties were evaluated. The quality of the experimental animal studies was assessed and was rated using Klimisch scores: score 1 (reliable without limitations), 2 (reliable with limitations), 3 (unreliable) or 4 (not assignable) [33]. Human data were also assessed, including a risk of bias analysis. Relevant parameters and tests for the identification of the substance as ED were identified based on the OECD test guideline 150 [34] and the review published by Manibusan and Touart (2017) [35]. A detailed description of all the studies considered and their quality assessment is available in the conclusion document written by ANSES ([36], in press). A total of 69 animal studies and 43 human epidemiological studies were considered and assessed for their reliability when evaluating the toxicological profile of the substance. From this selection, 29 animal studies and 23 human epidemiological studies were considered key in the assessment of the ED properties of the substance. Figure 1 presents the information flow diagram used for data search. The references, the reliability assessment and the type of studies used in this evaluation are available in Tables S2 and S3 of the supplementary material.

The relevant parameters have been grouped, as recommended in the guidance, into four categories: parameters measured in silico/in vitro, parameters measured in vivo that provide information on potential alteration of the endocrine function (referred as endocrine activity), parameters measured in vivo that provide information on adversity and indicative of estrogen, androgen, thyroid or steroidogenesis MoA (referred as EATS-mediated), parameters measured in vivo that are sensitive to, but not diagnostic on their own of EATS. The information was tabulated into lines of evidence. Epidemiological studies were not included and only used as supportive data. Each line of evidence was assessed based on quality and consistency of the effect along the studies. On a weight-of-evidence basis, each line was categorized into “sufficient evidence”, “insufficient evidence”, “no evidence” or “lack of data”. In addition, for each line, the indicated modality (Estrogen, Androgen, Steroidogenesis, Thyroid or not assignable to a specific modality) was specified. As proposed in the guidance, the lines of evidence were compiled to conclude whether an effect provided positive evidence of activity or adversity. The parameters assembled into lines of evidence are presented in the supplementary material—Table S4.

Following analysis of the evidence for both adversity and endocrine activity, a MoA analysis was performed. When an Adverse Outcome Pathway (AOP) was developed as part of the OECD AOP Development Program, the AOP was used as a tool to determine if CS_2_ was exerting an adverse outcome by an ED mechanism. In addition, the general knowledge on the plausible mechanism of adverse outcome have also been considered and their plausibility weighted in response to CS_2_ exposure.

From this work ([36], in press), it appears that most of the identified adverse effects could be related to endocrine related-MoA or could as well result from non-endocrine mediated MoAs. The analysis of the ED properties of CS_2_ therefore consisted in evaluating what is referred as specificity in the European guidelines [5]. As thyroid disruption is well described and because neurotoxicity and cardiotoxicity are the most relevant and better described adverse effects in humans, and also because they are known as potentially related to thyroid disruption, the publication focuses on evaluating the specificity of thyroid disruption for these two adverse effects.

## 3. Results and Discussion

### 3.1. Adverse Health Effects Induced by CS_2_ Exposure

Table 1 provides an overview of CS_2_-induced adverse effects characterized with sufficient evidence.

Two of these adverse effects are used as a basis of the current occupational exposure limit and may be related to the disruption of thyroid homeostasis: neurotoxicity and cardiovasular impairment [37,38,39]. Regarding thyroid disruption, it should be noted that there was no effect on thyroid weight or histopatological alterations following CS_2_ exposure.

Other adverse effects such as sperm morphology alterations, decreased ovary follicle count and skeletal malformations can also be associated with thyroid disruption [40,41,42,43], but will be discussed only briefly.

#### 3.1.1. Nervous System Effects Induced by Developmental or Adult Exposure to CS_2_

A total of five experimental studies investigated the potential neurotoxicity induced by CS_2_ exposure during gestation and/or lactation, either by the oral or inhalation route.

In a recent Extended One-Generation Reproductive Toxicity Study (EOGRTS) [44], performed according to OECD technical guidance 443, rats were exposed by gavage to 0, 1.2, 12 or 120 mg/kg bw/d CS_2_ in corn oil. This oral dose should correspond to about 10, 100 or 950 mg/m^3^, respectively, by inhalation [6]. F0 and F1-generation males and females were exposed 10 weeks before mating. In addition, females were exposed during gestation and lactation. In this study, several behaviors and parameters were measured in rats. The functional observational battery assessment, performed in animals of the F1-generation at postnatal days (PND) 63–75 showed a dose-related tendency to decrease in landing foot splay test, but was not reported as statistically significant in males or females. The physiological meaning of this tendency to decrease is, however, not clear since no other effects on motor activity, grip strength or auditory startle response were affected by the treatment.

Analyses of brain histopathology and morphometry showed, at the maximal dose of 120 mg/kg, a significant decrease in absolute brain weight (−5% to −10% compared to control) in adult males and females and in males of the F1 generation at PND 22. Similar effects were observed in F2 generation males at PND 21–23. In addition, a significant increase in the mean caudate putamen (striatum) width was noted in females at PND 21–22 or at PND 76–90 at 120 mg/kg. The striatum, a structure of the basal ganglia, receives afferent inputs from the cerebral cortex, thalamus and dopaminergic nuclei (substantia nigra and ventral tegmental area) and plays key roles in motor function, reward and also emotion and cognition [45]. A significant decrease in the thickness of the corpus callosum, the bundle of nerve fibers that connect the two hemispheres, was also observed in males at 120 mg/kg. Moreover, marked retinal atrophy evidenced by a loss of cell layers and considered as a sign of neurotoxicity was also described at this dose in the F1-generation at PND 89–95. It would have been interesting to conduct further analyses, including other behavioral tests, to determine whether these morphometric and neuroanatomical effects translate into changes in brain function.

In the four remaining studies conducted via inhalation, three studies reported developmental delays and neurobehavioral effects in offspring rats exposed in utero over one or two generations to CS_2_ at ≥0.03 mg/m^3^ [46,47,48]. The authors reported delayed eye opening and auditory function as well as impaired exploratory or motor activity in the open-field test in pups from 10 mg/m^3^. In Lehotzky et al. (1985), female rats were exposed by inhalation to 10, 700, or 2000 mg/m^3^ CS_2_ from gestational days 7 to 15, 6 h per day by inhalation [49]. Reduced mean pup weight, delayed eye opening and immature righting reflex were observed at PND 21 particularly at the two highest doses. In addition, the latency of the conditioned avoidance response was significantly prolonged for all doses of CS_2_ in pups. It may be noted that, at the two highest dose, a high mortality rate was reported in pups (35% at 700 mg/m^3^ and 50% at 2000 mg/m^3^) and in dams (33% at the maximal dose). These published studies suffer, however, from insufficiencies in the reporting experimental conditions and obtained data.

Overall, the EOGRTS provides some indications of neurotoxicity induced by gestational/lactational exposure of dams to oral CS_2_ as shown by the reduced brain weight and corpus callosum thickness, and enlargement of the striatum, a key brain area involved in motor control and learning. However, no significant effects were observed on motor function or coordination in adult animals. The studies by Lehotzky et al. (1985) and Tabacova’s group provided some evidence of delayed maturation and sensorimotor development in postnatal animals when exposed via inhalation. The comparison of these data raises questions about the impact of the route of exposure (oral gavage versus inhalation) on toxicokinetics and the doses used.

Neurotoxicity in adults has been investigated in four regulatory toxicity studies and in numerous academic studies.

There are three standard regulatory 90-day repeated-dose toxicity studies with CS_2_. Two strains of rats [50,51] and mice [52] were exposed to CS_2_ vapors at 158, 948, 2528 mg/m^3^ for 90 days. Brain absolute weight was decreased at ≥153 mg/m^3^ in male rats, at ≥948 mg/m^3^ in female rats and at 2528 mg/m^3^ in mice. Axonal swelling of nerve fibers of the ventral and lateral funiculi of the spinal cord for both sexes, segmental degeneration of fibers in the sciatic nerve were observed in both strain of rats and in mice at the top dose. In the oral gavage EOGRTS [44], absolute brain weight was significantly decreased in both sexes of the F0-generation at the maximal dose of 120 mg/kg (−6% and −5% versus male or female controls, respectively). In addition, marked retinal atrophy characterized by loss of cell layers was also described at this dose in the F0-generation and considered as a sign of neurotoxicity.

Academic studies also provided evidence of neuropathological and neurobehavioral consequence of the inhalation of CS_2_ following repeated exposure (i.e., 5–13 weeks) in adult rats or mice.

Inhibition of avoidance response, decreased response to a visual stimulus, hind-limb motor difficulties and gait abnormalities in adult rats were reported by several authors, at the lowest effective dose of 1251 mg/m^3^ [53,54,55,56,57,58,59,60]. Spatial learning and memory were assessed in Wang et al. (2017) using the Morris water maze tests. Rats exposed to ≥200 mg/kg CS_2_ by oral route displayed memory impairments [61].

Loss of hearing in rats and visual damage in monkeys (irreversible severe reduction in visual acuity with degeneration of retinal ganglion cells, axonal swelling of the optic nerve) were also reported [62,63,64]. With regards to neuropathology, several studies reported neuron axonal swelling in the peripheral as well as in the central nervous system, usually at 1580 mg/m^3^ onward [65,66,67,68,69]. The effect was accompanied by neurofilamentous accumulation [58,59,68,69] and myelin thinning [65]. In addition, an adverse effect on mating behavior (increased latency to mount and to ejaculation) was described in rats exposed for 10 weeks to CS_2_ at 1896 mg/m^3^ [70,71].

In humans, CS_2_ also targets the central and peripheral nervous systems. Polyneuropathy in workers was characterized by axonal loss, focal axonal swelling and neurofilamentary accumulation. Reduced nerve conduction velocity and impaired performance on psychomotor tests have been reported in workers [9]. Effects on the autonomic nervous system, vision and retinopathy were also observed. Effects on the nervous system were clear at concentrations ≥30 mg/m^3^ and already reported at exposure >3 mg/m^3^ [9,27,28]. In addition, impaired libido alteration in humans was reported in some studies at dose levels close to the current occupational exposure limit [15,72].

Overall, indication of neurotoxicity was evidenced in adult animals and in humans following repeated exposure to CS_2_.

#### 3.1.2. Cardiovascular Impairments

Evidence of cardiovascular alterations has been observed in rats and mice. Several in vivo studies have investigated the potential effect of CS_2_ on the cardiovascular system in adult animals. In the three standard 90-day repeated-dose toxicity studies in rats and mice [50,51,52], an increase in relative heart weight was observed at 2528 mg/m^3^ in both rat strains and in mice of both sexes. In the EOGRTS described above [44], heart relative weight was decreased in adults of F0 and F1 generations (−8% compared with controls).

Evidence for lipid disturbances was consistently reported in all 3 academic in vivo studies. Lipid disturbances in the vascular walls and in the blood serum, a key process in atheroma formation in humans, have been described in rats exposed to levels of 230 to 1700 mg/m^3^ after 12 to 15 months of exposure [73]. An increase in fatty arterial deposits was noted in mice exposed to CS_2_ by inhalation at 158, 1580 or 2528 mg/m^3^ for 1, 4, 8, 12, 16 or 20 weeks, 5 days per week [74]. Structural and functional changes (lumen distention, myocardial vessels attenuation, irregular thickening of the aorta wall and microscopic histological changes) have been described in rats exposed to 58 mg/m^3^ CS_2_ by Antov et al. (1985) [75].

Some human epidemiological studies have indicated severe cardiovascular disease, i.e., increased risk of coronary heart disease in workers exposed to CS_2_ in viscose rayon plants [76,77]. This may be due to an increase in LDL cholesterol and a decrease in high-density lipoprotein (HDL) cholesterol, which have been reported in several cohorts. Ischemic findings have also noted at low exposure levels, around 15 mg/m^3^ [22].

### 3.2. Disrupting Effect of CS_2_ on Thyroid Function

#### 3.2.1. Molecular Evidence

Based on the analysis of the potential endocrine activities of CS_2_, its effect on blood T4 levels was considered the most likely endocrine disruption.

No reliable in silico/in vitro studies have been identified on CS_2_. However, it should be noted that there is evidence of potential disruption of thyroid homeostasis by one of the known metabolites of CS_2_, thiourea. This metabolite has been shown to inhibit the activity of thyroid peroxidase (TPO), a key enzyme in thyroid hormone (TH) biosynthesis, in rats or in TPO-transfected *E. coli* in in vitro screening studies [32].

**Figure 2:**
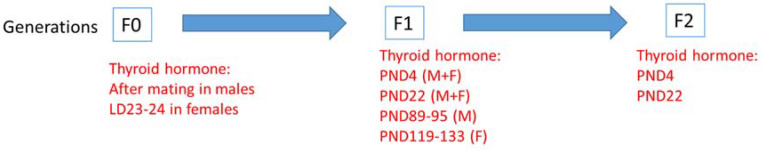
Simplified overview of the experimental design of the EOGRTS: TH measurements [44]. PND: postnatal day; LD: lactating day, M: males; F: females.

In the EOGRTS discribed above [44], TH concentrations were measured at different time points in the F0, F1 and F2 generations. An overview of the experimental design and sample time points is provided in Figure 2.

A decrease in serum total T4 concentration was noted in F0-generation males and females (−49% and −31% compared to controls, respectively) and in F1 males at PND 89–95 (by 26% compared to controls) at 120 mg/kg bw/day. At other time-points and generations, total T4 levels were not affected by CS_2_ exposure. There was no effect on triiodothyonine or thyroid-stimulating hormone levels in the study.

These results are consistent with the observations made in adult male rabbits by Van Stee et al. (1986) [78]. In this study, rabbits were exposed for 12 weeks to CS_2_ at 300 ppm in the air (i.e., a similar range of exposure to the highest dose in the rat gavage study described above), to thiourea at 208 mg/kg bw or to a 2% cholesterol diet with or without inclusion of T4 in the diet. Although caution should be exercised in the interpretation of these results due to the very small number of animals per group, some effects of CS_2_, thiourea or cholesterol on T4 levels were demonstrated.

Although not consistenly seen in epidemiological studies, an effect of CS_2_ on T4 concentration was also reported in several human cohorts [20,21,22,23], even at low occupational exposure levels around 15 mg/m^3^ [22], supporting potential human relevance of experimental animal data for this effect.

It should be noted that there were no data relevant to the assessment of ED effects of CS_2_ on wildlife.

Overall, the in vitro and in vivo data strongly suggest that CS_2_ exposure results in a decrease in T4 level, attesting to an endocrine activity on the thyroid modality. Interestingly, the key characteristic approach proposed by La Merill et al. (2020) would lead to the conclusion that CS_2_ can alter hormone distribution or circulating hormone levels [26].

#### 3.2.2. Thyroid Disruption as a Potential Early Key Events of Neurotoxicity

Given the crucial role of THs in the development and normal function of the central nervous system, it can be assumed that thyroid disruption plays a role in the neurotoxic effects induced by CS_2_ exposure. The key role of THs on brain development and neural adult function is particularly clear for processes underlying cognitive function (learning and memory), sensorimotor development and motor behavior/locomotor activity [40].

In the context of developing AOPs linking molecular initiating events targeting the thyroid system to neurotoxicity, AOP42 [79] may be considered. This AOP links inhibition of TPO activity, as a molecular initiating event, to adverse neurodevelopmental outcomes, particularly on hippocampal anatomy and function, and decreased cognitive function. Interestingly, some of the key events in this AOP were observed with CS_2_. Thiourea, one of the metabolites of CS_2_, has been reported to be a TPO inhibitor. The inhibition of TPO results in a decrease in TH synthesis and subsequent reduction in circulating TH concentrations. As described in Section 3.2.1, a decrease in T4 concentration has been observed in several studies and in several species [44,78]. Whether such hormonal deficiency could have triggered alterations in hippocampal formation and associated cognitive functions remains to be investigated with CS_2_.

TH disruption can also induce sensorimotor alterations, such as the those observed in some studies following CS_2_ exposure during development [46,47,48,49]. To date, there is no AOP linking TPO inhibition or thyroid disruption during development to adverse motor dysfunction, but a causal relationship remains plausible.

Impaired spatial learning and memory as well as motor dysfunction have been observed in adult rats exposed to CS_2_ [57,61] as well as in workers [9]. Although no investigation has been conducted to establish a link between disruption of the thyroid function and cognitive or motor deficiencies in CS_2_ exposed adults, a link cannot be ruled out.

#### 3.2.3. Thyroid Disruption as a Potential Early Key Events of Cardiovascular Disease

**Figure 3:**
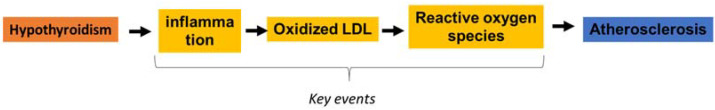
Postulated MoA for CS_2_: decreased TH levels and subsequent adverse atherosclerosis, adapted from Duntas et al. (2018).

There is also a well-established link between low serum TH and increased LDL cholesterol [80,81]. Duntas et al. (2018) proposed a physiological pathway between clinical hypothyroidism and potential atherosclerosis formation (Figure 3). Elevated serum LDL cholesterol is a major risk factor for coronary heart disease because LDL is the predominant atherogenic lipoprotein. Interestingly, elevated LDL cholesterol and ischemic coronary disease have been observed in workers exposed to CS_2_. Thus, a plausible link between the observed effect on T4 levels and coronary heart disease can be hypothesized. It should be noted, however, that in rabbits, exposure to 950 mg/m^3^ of CS_2_ or to its metabolite, thiourea, despite the reduction in the serum T4 levels, did not induce an atherogenic response or any other sign of vascular damage discernible by gross examination or light microscopy.

### 3.3. Which MoA(s) Are Responsible for Neurotoxicity?

#### 3.3.1. Other Potential Molecular Events Involved in Neurotoxicity

There are several hypotheses regarding the neurotoxic MoA of CS_2_. It has been postulated that the axonal degeneration and neurofilament accumulation related to the central-peripheral neuropathy results from the metabolism of the substance to reactive adduct-forming intermediates, i.e., dithiocarbamates. CS_2_ forms dithiocarbamates by combination with amino acids, sulfhydryl, glutathione or cysteine. Dithiocarbamates have been detected in animals and humans [29]. These metabolites are electrophilic compounds that react with nucleophilic proteins on neurofilaments to cause protein cross-linking. The mass of covalently cross-linked neurofilaments can impede axonal transport (i.e., at the nodes of Ranvier) resulting in axonal swelling and degeneration. Neurofilamentous cross-linking was observed upon air exposure at 158 mg/m^3^ CS_2_ [69]. After oral exposure of rats for 12 weeks, changes of neurofilament cytoskeleton protein content in rat cerebrum and altered neurofilament content in the spinal cord were observed in 2 studies at ≥300 mg/kg bw/day CS_2_ [58,59].

CS_2_ and its dithiocarbamate metabolites have also been shown to react with amino acids and to chelate essential metals (e.g., Zn^++^ and Cu^++^), affecting important enzymes, such as dopamine-β-hydroxylase (DBH), alkaline phosphatase, monoamine-oxidase [29,82,83]. In this context, the reaction of CS_2_ with neuronal amines and metal/enzyme complexes has been assumed to be involved in neurotoxicity.

DBH, a mono-oxygenase with a copper in its site of action, converts dopamine to norepinephrine. The increase in dopamine and the concomitant decrease in norepinephrine, resulting from the inhibition of DBH by CS_2_, can influence the homeostasis of various brain functions. The morphometric changes observed in the striatum of the F1 generation in the EOGRTS may support the hypothesis of catecholamine disruption, as this brain region receives input from dopaminergic nuclei. The effect on male behavior could also be related to catecholamine disruption. Furthermore, one possible consequence of catecholamine disruption, among others, could be an effect of CS_2_ on thyrotropin-releasing hormone neurons, regulating the thyroid axis, since these neurons are known to be tightly regulated by noradrenergic neurons [84].

CS_2_ can interact with sulfhydryl- and amino groups of proteins and thus, the nucleophilic group of enzymes. Some authors have suggested that CS_2_ may interfere with nitric oxide (NO) synthase and NO synthesis [85]. NO serves as a neurotransmitter in the central and peripheral nervous system and is produced by endothelial cells [86]. NO also plays a role in cardiovascular homeostasis [87]. Guo et al. (2008) showed that exposure of rats to CS_2_ by inhalation at 0, 50, 250 and 1250 mg/m^3^ for 2 months reduced constitutive NO synthase (NOS) activity and neuronal NOS mRNA levels, and increased induced NOS mRNA levels in the hippocampus [88]. The authors suggested that these effects may underlie the impairments in spatial learning and memory in the same study in exposed rats. However, the formation of reactive oxygen species (ROS), as shown below, can also reduce the concentration of NO by consuming it directly or by damaging the NOS structure.

Oxidative stress is associated with CS_2_ induced polyneuropathy. Wang et al. (2017) [61] reported impaired cognitive performance of adult rats after oral 20-day exposure to 200, 400 or 600 mg/kg. It was observed that extensive oxidative stress was induced by CS_2_ and that mitochondria-dependent apoptosis pathways were implicated in neuronal loss in the hippocampus. In a previous study, and at similar dose levels, Wang et al. (2016) [89] exposed adult rats to CS_2_ for 6 weeks. The authors showed that CS_2_ exposure was associated with the activation in nervous tissues of the nuclear factor 2-related factor 2, which is involved in the protection of cells against oxidative stress. Sun et al. (2009) [90] observed that in the cerebral cortex, hippocampus, spinal cord and serum of rats after 0, 2, 4, 8 and 12 weeks of CS_2_ administration at 1250 mg/m^3^, ROS and malondialdehyde levels were induced, with a concomitant decrease in antioxidant status, i.e., GSH content. A significant correlation between lipid peroxidation and gait abnormalities was observed as symptoms developed. Some human epidemiological studies also reported oxidative stress. In a human observational study, workers exposed to CS_2_ had higher levels of malondialdehyde and reduced levels of antioxidative enzymes compared to controls [91]. Jian and Hu (2000) [92] reported that, compared with control subjects, serum cuprozinc-superoxide dismutase levels and serum malondialdehyde levels were increased in a concentration and time-dependent manner in the CS_2_ exposed worker group.

#### 3.3.2. Is It Possible to Distinguish the Different MoAs Underlying CS_2_ Neurotoxicity?

The available data suggest that CS_2_ may have more than one MoA that may be related to both endocrine and non-endocrine pathways. In this case, the European guidance recommends considering which MoA would provide the most compelling evidence [5]. Behavioral changes such as sensorimotor or cognitive impairments have been observed after CS_2_ exposure and may be related to thyroid disruption.

On the one hand, the biological plausibility of the relationship between thyroid disruption and developmental cognitive effects or sensorimotor deficits is well established. In the case of CS_2_, there is evidence of sensorimotor impairments in pups exposed in utero (e.g., Lehotzky [49]). Nevertheless, the study had some limitations that led to some uncertainties. In addition, no assessment of learning and memory was undertaken in the EOGRTS or in academic studies after exposure to CS_2_ during development_,_ as noted above in Section 3.1.1. Therefore, no data are available to support this hypothesis. In contrast, neurobehavioral changes following adult exposure to CS_2_ in animals or humans have been widely reported. However, the link between moderate thyroid impairment and neurocognitive or motor effects in adult is less well established, which also makes the biological plausibility of the causal relationship between decreased T4 levels and neurocognitive impairment in adults uncertain.

On the other hand, it is plausible that the cognitive and sensorimotor effects are biologically related to a general toxic effect of CS_2_ in the brain. There is strong evidence that CS_2_ induced brain toxicity. Absolute brain weight was decreased at dose levels as low as 158 mg/m^3^ in the 90-day inhalation study in rats. In the oral EOGRTS, a decrease in brain weight was also observed at 120 mg/kg but not at 12 mg/kg (corresponding to dose levels about 100 and 950 mg/m^3^, respectively). Brain histopathological findings (axonal swelling and segmental degeneration of nerve fibers) were noted at 2528 mg/m^3^ in the 90-day inhalation studies in rats and mice. In academic studies, axonal swelling was generally detected at concentrations of 1580 mg/m^3^.

Temporality is also an important parameter for highlighting early ED-mediated events potentially arising from general toxicity. In the EOGRTS, a decrease in T4 levels was noted after at least 10 weeks of exposure in the F0 or F1 generations. Changes in T4 level were not observed at PND 4 or PND 22 in the F1 or F2 generations, whereas morphological alteration of the brain and toxicity were already noted in F1 and F2 pups at PND 22. This suggests that the toxic effects on the brain may precede the thyroid disruption. Nevertheless, the observed effects on T4 concentration in the F1 generation may result from in utero exposure to CS_2_ and/or maternal thyroid disruption, as suggested by the decreased T4 concentrations in the serum of lactating females of the F0 generation. Sampling at additional time points, i.e., during gestation, would have been needed to draw a firm conclusion.

Data available do not suggest that CS_2_ acts more specifically on the neuroendocrine system rather than on the whole brain. Hypothalamic cells, particularly those near the median eminence, are not protected by the classical non-fenestrated blood–brain barrier and these cells may be more exposed than other neural cell types during development. However, there is no evidence showing that neuroendocrine cells (e.g., in the hypothalamus) might be more sensitive to the cytotoxic effect of CS_2_ than other cerebral tissues. Regarding the potential tissue targets underlying the observed cognitive and motor effects, there is no evidence that the brain regions involved in these functions (hippocampus, striatum, etc.) would be more sensitive to the toxic action of CS_2_ than other brain areas. Therefore, there is no evidence of a specific endocrine-mediated MoA.

Based on the weight of evidence, CS_2_-induced behavioral impairments are not based specifically and exclusively on thyroid disruption. Indeed, too many uncertainties remain to assert that the adverse effects on neural development and function are a consequence of thyroid disruption, and there are too many gaps in the data to refer to any of the validated AOP.

Moreover, other molecular events such as alteration of catecholamine homeostasis, ROS induction, NO synthase inhibition, neurofilament cross-linking are involved in the neurotoxic potential of the substance. Some of these molecular events can be considered as part of both ED and non-ED MoA pathways. For example, hypothyroidism can induce oxidative stress in cells, supporting the ED pathway hypothesis. It is even more difficult to conclude because the molecular pathway, the dose levels and the temporal concordance between all these potential molecular events are unknown. Nevertheless, it should be noted that adduct formation is well established even at low dose levels (i.e., 153 mg/m^3^) most likely in the absence of a decrease in T4 level (no effect in the EOGRTS at 12 mg/kg, extrapolated to 100 mg/m^3^ by inhalation) which would support the direct toxic action of CS_2_.

Overall, thyroid disruption does not appear to be the initiating event for neurotoxicity given the available data on CS_2_ toxicity. Based on these data, it seems more plausible that both thyroid disruption and behavioral impairments are consequences of direct CS_2_ toxicity to the brain. Thyroid disruption may then be a nonspecific secondary ED mechanism resulting from the systemic CS_2_ toxicity responsible for the neurotoxicity.

### 3.4. Which MoA(s) Are Responsible for Cardiotoxicity?

#### 3.4.1. Other Potential Molecular Events Involved in the Alteration of the Cardiovascular System

Excessive oxidative damage may also be involved in the cardiovascular toxicity of CS_2_. Laurman et al. (1989) [93] found that CS_2_ interacts in vitro with LDL, resulting in increased electrophoretic mobility of particles, due to a decrease in free amino groups of apolipoprotein B-100. CS_2_ modification decreases the ability of LDL to down-regulate sterol synthesis and to stimulate cholesterol esterification in fibroblasts. Wronska-Nofer et al. (1996) [94] also found that, in vitro, CS_2_ can oxidize LDL and increase its cytotoxicity. Wronska-Nofer et al. (2002) studied the role of oxidative stress in the premature development of atheroma in men chronically exposed to CS_2_ and diagnosed with atherosclerosis [95]. The levels of thiobarbituric reactive substances (TBARS), measured as a marker of lipid peroxidation, were elevated in exposed group compared with healthy unexposed adults. Although the exact chemical reactions between CS_2_ and LDL in vivo are not fully elucidated, CS_2_-induced chemical changes, such as oxidation of LDL, are closely associated with increased LDL uptake by macrophages and the development of arterial fatty streaks. Taken together, these data support the idea that CS_2_ may alter cholesterol homeostasis through perturbations in oxidative or protein metabolism.

#### 3.4.2. Is It Possible to Distinguish between the Different MoA(s) Responsible for the Damage to the Cardiovascular System?

With respect to the effects on the cardiovascular system, there are many gaps in the understanding of the full sequence of key events linking hypothyroxinemia and hypercholesterolemia and leading to atherogenesis.

A direct interaction of the substance with LDL cholesterol has been suggested and may be a non-ED MoA. In addition, oxidative stress may also be a key factor in the development of cardiovascular disease without any thyroid disruption. A toxic action of CS_2_ on the heart was observed in the standard regulatory studies at 2528 mg/m^3^ by inhalation in rats or mice and at 120 mg/kg in the EOGRTS in F0 and F1 generation males.

As noted for neurotoxicity, dose concordance did not help distinguish potential MoAs because both T4 decrease and cardiotoxicity (e.g., heart weight decrease) occurred at the same dose level, so it was not possible to determine which one of these effects is more sensitive to CS_2_.

In conclusion, the available data on CS_2_ cardiotoxicity suggests that thyroid disruption and atherogenicity are consequences of direct CS_2_ toxicity on cardiovascular toxicity.

## 4. Conclusions

Following the assessment of the potential ED properties of CS_2_, a decrease in T4 was identified with sufficient evidence. Adverse effects known to be potentially linked to thyroid disruption were observed with CS_2_, in particular, neurotoxicity and cardiotoxicity. Analysis of the possible causes leading to the observed adverse effects suggests the involvement of several complex ED and/or non-ED MoAs.

The regulatory definition used to identify an ED states that the adverse effect must be a consequence of the ED MoA. This relationship is normally established on the basis of broad general knowledge in the field.

When different MoAs are identified, the EFSA/ECHA guidance document [5] describes different criteria to discriminate these MoA as the most plausible ones. Dose and temporal concordance are important elements that should be considered when determining the relationships between the key events. Several other elements need to be considered. Essentiality is a consideration in assessing whether the MoA is essential for the adverse effect to occur. Consistency reminds that the MoA should be consistently reported among studies, species, strains and systems. Analogy consists in appreciating if the causal relationship between the two events has been described for other substances. In the European guidance, the question of deciding whether the adverse effect is mediated through an endocrine rather than a non-endocrine MoA is referred as specificity. Specificity involves comparing different MoAs to determine which of them should be considered as the initiating event for the adverse effects of interest.

The CS_2_ case illustrates the difficulty of finding studies designed to address these elements with certainty. A thorough analysis of the dose levels leading to thyroid disruption and neurotoxicity, and of the temporality of these effects (i.e., which effect precede the other) showed that they occurred concomitantly and simultaneously within the limits of the experimental design. It may be noted that additional time points for the dosage of TH would have been necessary to be able to accurately determine the sequence of events leading to the adverse effect. For example, it would have been important to measure TH in the dams during gestation, to determine whether altered behavior of the pups could be related to maternal thyroid disruption as it is often the case. In addition, a specific design including additional dose levels might have been useful to identify whether thyroid disruption could be observed at dose levels below brain toxicity. Standard regulatory studies are designed to report toxic effects without considering distinguishing potential ED from non-ED MoAs. Nevertheless, in the CS_2_ case, it cannot be excluded that the neural effects precede thyroid disruption since both effects were observed concomitantly, weakening the hypothesis of thyroid disruption as the initiating event of the neural impairment.

As described above, the impact of thyroid disruption on neurotoxicity is well known and described in response to exposure to different substances. The analogy is therefore fulfilled. In addition, thyroid disruption has been consistently reported when assessed in animals after exposure to CS_2_. There were no robust data available to study the essentiality of thyroid disruption. Finally, specificity, i.e., considering if the MoA leading to the adverse effects studied was specifically ED-related, was an important element in the conclusion of our case. Indeed, the high toxic potential of the substance, through adducts formed by reactive metabolites, is well known. In the study of Lehotzky [49], no excessive systemic toxicity such as changes in body weight gain or effects on survival were noted in the regulatory toxicity studies up to the highest dose. However, CS_2_ is neurotoxic and brain effects were noted in the analyzed studies. Direct brain or heart toxicities rather than a specific ED MoA were considered as responsible for the induced effects on the nervous and cardiovascular systems.

Finally, as mentioned above regarding temporality of the different events, there is no evidence that the decrease in T4 precedes non-endocrine systemic toxicity. Thus, thyroid disruption appears rather as a nonspecific secondary ED mechanism of systemic toxicity. The same pattern applies to other adverse effects reported after CS_2_ exposure: sperm effects, decreased ovary follicle count and skeletal malformations (not detailed here). Therefore, both the data and the general biological knowledge support the conclusion that CS_2_ is not an ED.

Data on the mechanism and molecular initiating events are not routinely available in standard regulatory studies. Even in the case of a data-rich substance, such as CS_2_, in vitro data were insufficient to establish the AOP leading to neurotoxicity. In addition, some important adverse effects, such as cognitive function assessment in developing animals, are not required in standard regulatory tests, even though it is likely to occur after exposure to CS_2_. This example raises questions about (i) the ability of available techniques required in toxicology to address such a complex issue with certainty and (ii) the need to revise OECD technical guidance as a potential way forward.

## Figures and Tables

**Table 1:** Overview of adversities potentially related to an ED property of CS_2_.

Parameters	Effect
Sperm morphology abnormalities	↑
Sperm number	↓
Time to mating	↑
Ovary primary follicles	↓
Malformations (visceral and skeletal)	↑
Embryonic or fetal deaths	↑
Brain histopathology	Altered
Brain morphometry	Altered
Behavior	Altered
Retinal atrophy	↑
Carbohydrate level	↑
Low density lipoprotein (LDL) cholesterol	↑
Coronary histopathology	Altered

↑: Increased, ↓: decreased.

## Supplementary Materials

The following supporting information can be downloaded at: https://www.mdpi.com/article/10.3390/ijms23063153/s1.

## Abbreviations

ANSES	Agence Nationale de Sécurité Sanitaire de l’alimentation, de l’environnement et du travail (French Agency for Food, Environmental and Occupational Health & Safety)
AOP	Adverse outcome pathway
CS_2_	Carbon disulfide
DBH	Dopamine-β-hydroxylase
EATS	Estrogen, Androgen, Steroidogenesis, Thyroid
ECHA	European chemical agency
ED	Endocrine disrupting
EFSA	European food safety authority
EOGRTS	Extended One-Generation Reproductive Toxicity Study
HDL	High-density lipoprotein
LDL	Low-density lipoprotein
LD	Lactating day
MoA	Mode of action
NO	Nitric oxide
NOS	Nitric oxide synthase
OECD	Organisation for Economic Co-operation and Development
PND	Postnatal day
REACh	Regulation (EC) No 1907/2006 of 18 December 2006 concerning the registration, evaluation, authorisation and restriction of chemicals;
ROS	Reactive oxygen species
TH	Thyroid hormone
T4	Thyroxin
TPO	Thyroid peroxidase
