# Supplementary material for: How to Differentiate General Toxicity-Related Endocrine Effects from Endocrine Disruption: Systematic Review of Carbon Disulfide Data
Source: Int J Mol Sci. 2022 Mar 15;23(6):3153. doi: 10.3390/ijms23063153 (PMC8952789; doi:10.3390/ijms23063153)
Supplement: Supplementary file 1 [file ijms-23-03153-s001.zip › ijms-1615026-supplementary.pdf]

Supplementary Materials

Table S1: Inclusion and exclusion criteria for literature search.

Publication type	IN	Primary research studies
	OUT	Secondary studies (e.g. editorials, conference)
Language	IN	English
	OUT	Other languages
Study design	IN	Human experimental volunteer studies<br>Cohort studies<br>Cross-sectional studies<br>Case-control studies<br>Experimental animal studies
Population	IN	All mammalian animals<br>Adult healthy male and female volunteers, men and women occupationally exposed to CS2
Exposure	IN	All route of exposure relevant to occupational exposure
	OUT	mixture
Time	IN	From January 2013 to December 2019
Outcome	IN	Human health
	OUT	Environment

Table S2: Reliability, study type and references of *in vivo* studies used in the assessment of carbon disulfide ED properties.

**Reliability**	**Study type**	**Route of exposure**	**Reference**
K4	Non-guideline 4-month female rat toxicity study	Inhalation	Agadzhanova, A. A. '[Industrial hygiene problems of women in the manufacture of viscose fiber]'. *Gigiena Truda I Professional'nye Zabolevaniia*, no. 4 (1978): 10–13.
K4	Non-guideline 6-month study in rats	Inhalation	Antov, G., B. Kazakova, M. Spasovski, K. Zaikov, M. Parlapanova, S. Pavlova, and M. Stefanova. 'Effect of Carbon Disulphide on the Cardiovascular System'. *Journal of Hygiene, Epidemiology, Microbiology, and Immunology* 29, no. 4 (1985): 329–35.
K4	Non-guideline prenatal developmental toxicity study	Inhalation	Beliles, R.P, Brusick, D.J, and Mecler, F.J. 'Teratogenic-Mutagenic Risk of Workplace Contaminants: Trichloroethylene, Perchloroethylene and Carbon Disulphide.' ; Final contract report. U.S. Department of Health, Education and Welfare, Public Health Service, Center for Disease Control, NIOSH, Cincinnati, Ohio, 225 pages. 1980. Available online: https://www.cdc.gov/niosh/nioshtic-

			2/00113556.html (Assessed on January 31 2022)
K4	10-day non-guideline toxicity study	Inhalation	Caroldi, S., J. Jarvis, and L. Magos. 'Carbon Disulphide Exposure Affects the Response of Rat Adrenal Medulla to Hypothermia and Hypoglycaemia'. *British Journal of Pharmacology* 84, no. 2 (1985): 357–63. https://doi.org/10.1111/j.1476-5381.1985.tb12920.x.
K2	Non-guideline male rat testicular toxicity study	Inhalation	Gao, Yuan, Shasha Wang, Anji Yi, Ruirui Kou, Keqin Xie, and Fuyong Song. 'Activation of Lysosomal Degradative Pathway in Spinal Cord Tissues of Carbon Disulfide-Treated Rats'. *Chemico-Biological Interactions* 219 (2014): 76–82. https://doi.org/10.1016/j.cbi.2014.05.016.
K2	Non-guideline male rat testicular toxicity study	Inhalation	Guo, Yinsheng, Jiajia Ji, Wei Wang, Yu Dong, Zhen Zhang, Yijun Zhou, Guoyuan Chen, and Jinquan Cheng. 'Role of Endoplasmic Reticulum Apoptotic Pathway in Testicular Sertoli Cells Injury Induced by Carbon Disulfide'. *Chemosphere* 132 (2015): 70–78. https://doi.org/10.1016/j.chemosphere.2015.02.058.
K4	Non-guideline prenatal developmental toxicity study	Inhalation	Hardin, B. D, Bond, G. P., Sikor, M. R, Andrew, F. D, Beliles, R. P, and Niemeir, R. W. 'Testing of Selected Workplace Chemicals for Teratogenic Potential'. *Scand. J. Work Environ. Health.* 7, no. 4 (1981): 66–75.
K2	Non-guideline male rat toxicity study	Inhalation	Huang, Xiaoyu, Yijun Zhou, Jiying Ma, Ning Wang, Zhen Zhang, Jiajia Ji, Qing Ding, and Guoyuan Chen. 'Nitric Oxide Mediated Effects on Reproductive Toxicity Caused by Carbon Disulfide in Male Rats'. *Environmental Toxicology and Pharmacology* 34, no. 3 (2012): 679–87. https://doi.org/10.1016/j.etap.2012.10.001.
K4	Prenatal developmental toxicity study (Similar to OECD 414)	Oral: gavage	Jones-Price, C., Tyl, Marr, and Kimmel. 'Teratologic Evaluation of Carbon Disulfide (CAS No. 75-15-0) Administered to CD Rats on Gestational Days 6 through 15.' National Center for Toxicological Research, Jefferson AR. Govt. Reports Announcements and Index, Issue 15. NTIS PB 84-192343, 1984. https://ntrl.ntis.gov/NTRL/dashboard/searchResults/titleDetail/PB84192343.xhtml (accessed on 31 January 2022).
K4	Prenatal developmental toxicity study (Similar to OECD 414)	Oral: gavage	Jones-Price. 'Teratologic Evaluation of Carbon Disulfide (CAS No. 75-15-0) Administered to New Zealand White Rabbits on Gestational Days 6 through 15.' National Center for Toxicological Research, Jefferson AR. Govt. Reports Announcements and Index, Issue 15. NTIS PB 84-192350. Cited from ATSDR [29]

K4	Non-guideline male toxicity study	Inhalation	Kumar, S., K. G. Patel, A. K. Gautam, K. Agarwal, B. A. Shah, and H. N. Saiyed. 'Detection of Germ Cell Genotoxic Potential of Carbon Disulphide Using Sperm Head Shape Abnormality Test'. *Human & Experimental Toxicology* 18, no. 12 (1999): 731–34. https://doi.org/10.1191/096032799678839608.
K3	Non-guideline prenatal behavioural toxicity sudy	Inhalation	Lehotsky, K., Szeberenyi J.M, Ungvary, G., and Kiss, A. 'Behavioural Effects of Prenatal Exposure to Carbon Disulfide and to Aromatol in Rats.' *Arch Toxicol Suppl* 8 (1985): 442–46.
K4	Non-guideline mice toxicity study	Inhalation	Lewis, J. G., D. G. Graham, W. M. Valentine, R. W. Morris, D. L. Morgan, and R. C. Sills. 'Exposure of C57BL/6 Mice to Carbon Disulfide Induces Early Lesions of Atherosclerosis and Enhances Arterial Fatty Deposits Induced by a High Fat Diet'. *Toxicological Sciences: An Official Journal of the Society of Toxicology* 49, no. 1 (1999): 124–32. https://doi.org/10.1093/toxsci/49.1.124.
K4	Non-guideline male toxicity study	Intraperitoneal	Patel, K. G., P. C. Yadav, C. B. Pandya, and H. N. Saiyed. 'Male Exposure Mediated Adverse Reproductive Outcomes in Carbon Disulphide Exposed Rayon Workers'. *Journal of Environmental Biology* 25, no. 4 (2004): 413–18.
K2	Prenatal developmental toxicity study (Similar to OECD 414)	Inhalation	Saillenfait, A. M., P. Bonnet, and J. de Ceaurriz. 'Effects of Inhalation Exposure to Carbon Disulfide and Its Combination with Hydrogen Sulfide on Embryonal and Fetal Development in Rats'. *Toxicology Letters* 48, no. 1 (1989): 57–66. https://doi.org/10.1016/0378-4274(89)90186-0.
K4	Non-guideline 20-week study in monkeys	Inhalation	Sperlingová, I., V. Kujalová, and E. Frantík. 'Chronic Carbon Disulfide Exposure and Impaired Glucose Tolerance'. *Environmental Research* 29, no. 1 (1982): 151–59. c9.
K3	Non-guideline reproductive toxicity studies	Inhalation	Tabacova S, Hinkova L, and Balabaeva L. 'Carbon Disulphide Tetratogenicity and Postnataleffects in Rat.' *Toxicol Lett* 2 (1978): 252–54.
K4	Non-guideline reproductive toxicity studies	Inhalation	Tabacova, S., and L. Balabaeva. 'Subtle Consequences of Prenatal Exposure to Low Carbon Disulphide Levels'. *Archives of Toxicology. Supplement. = Archiv Fur Toxikologie. Supplement* 4 (1980): 252–54. https://doi.org/10.1007/978-3-642-67729-8_51.
K3	Non-guideline reproductive toxicity studies	Inhalation	Tabacova, S., B. Nikiforov, and L. Balabaeva. 'Carbon Disulphide Intrauterine Sensitization'. *Journal of Applied Toxicology: JAT* 3, no. 5 (1983): 223–29. https://doi.org/10.1002/jat.2550030502.

K2	Non-guideline male reproductive toxicity study	Inhalation	Tepe, S. J., and H. Zenick. 'The Effects of Carbon Disulfide on the Reproductive System of the Male Rat'. *Toxicology* 32, no. 1 (July 1984): 47–56. https://doi.org/10.1016/0300-483x(84)90033-7.
K2	90-day toxicity study in rats (similar to OECD 413)	Inhalation	Unpublished report. '90-Day Vapor Inhalation Toxicity Study of Carbon Disulfide in Fischer 344 Rats'. Study report, 1983a. Summary available online: https://echa.europa.eu/fr/registration-dossier/-/registered-dossier/14292/7/6/3 (accessed on 31 January 2022).
K2	90-day toxicity study in rats (similar to OECD 413)	Inhalation	Unpublished report. '90-Day Vapor Inhalation Toxicity Study of Carbon Disulfide in Sprague-Dawley Rats'. Study report, 1983b. Summary available online: https://echa.europa.eu/fr/registration-dossier/-/registered-dossier/14292/7/6/3/?documentUUID=e8305a96-98fa-4f42-9f32-361e5757c560 (accessed on 31 January 2022).
K2	90-day toxicity study in rats (similar to OECD 413)	Inhalation	Unpublished report. '90-Day Vapor Inhalation Toxicity Study of Carbon Disulfide in B6C3F1 Mice'. Study report, 1983c. Summary available online: https://echa.europa.eu/fr/registration-dossier/-/registered-dossier/14292/7/6/3/?documentUUID=40e5a301-2d25-4ccd-ac73-fc323334ede5 (accessed on 31 January 2022).
K2	Prenatal developmental toxicity study (Similar to OECD 414)	Inhalation	Unpublished report. 'Developmental Inhalation Toxicity Study of Carbon Disulfide in the New Zealand White Rabbit.' Pathology Associates, Inc. 1991. Summary available online: https://echa.europa.eu/fr/registration-dossier/-/registered-dossier/14292/7/9/3/?documentUUID=64f15538-168f-4dfb-85d9-cd2643156c78 (Assessed on 31 January 2022)
K2	Non-guideline female one-generation reproductive and developmental toxicity study	Oral: gavage	Unpublished report. 'Extended One Generation Reproductive Toxicity Study of carbon disulfide by Oral Gavage in Rats'. Unpublished study report, 2019. Available online: https://echa.europa.eu/fr/registration-dossier/-/registered-dossier/14292/7/9/2 (accessed on 31 January 2022).
K2	Extended-one generation reproductive toxicity study	Inhalation	Unpublished report 'One-Generation Reproductive and Developmental Toxicity Study'. Unpublished study report. 1992. Summary available online: https://echa.europa.eu/fr/registration-dossier/-/registered-

	(according to OECD 443)		dossier/14292/7/9/2/?documentUUID=c5ffc8a0-6d10-4767-a846-f849837871bf (Assessed on 31 January 2022).
K4	Non-guideline toxicity study in rabbits	Inhalation	Van Stee, E. W., J. E. Simmons, R. A. Sloane, M. P. Moorman, B. Adkins, and B. Y. Cockrell. 'Failure of Carbon Disulfide and Levothyroxine to Modify the Cardiovascular Response of Rabbits to a High-Cholesterol Diet'. *Toxicology* 40, no. 1 (1986): 45–58. https://doi.org/10.1016/0300-483x(86)90045-4.
K4	Non-guideline toxicity study in rats	Inhalation	Wrońska-Nofer, T., S. Szendzikowski, and M. Obrebska-Parke. 'Influence of Chronic Carbon Disulphide Intoxication on the Development of Experimental Atherosclerosis in Rats'. *British Journal of Industrial Medicine* 37, no. 4 (1980): 387–93. https://doi.org/10.1136/oem.37.4.387.
K2	Non-guideline male toxicity study	Inhalation	Zenick, H., K. Blackburn, E. Hope, and D. Baldwin. 'An Evaluation of the Copulatory, Endocrinologic, and Spermatotoxic Effects of Carbon Disulfide in the Rat'. *Toxicology and Applied Pharmacology* 73, no. 2 (1984): 275–83. https://doi.org/10.1016/0041-008x(84)90333-8.

Table S3: References of epidemiological human studies used in the assessment of CS_2 ED properties.

Cai, S. X., and Y. S. Bao. 'Placental Transfer, Secretion into Mother Milk of Carbon Disulphide and the Effects on Maternal Function of Female Viscose Rayon Workers'. *Industrial Health* 19, no. 1 (1981): 15–29. https://doi.org/10.2486/indhealth.19.15.
Cavalleri, A. 'Serum Thyroxine in the Early Diagnosis of Carbon Disulfide Poisoning'. *Archives of Environmental Health* 30, no. 2 (1975): 85–87. https://doi.org/10.1080/00039896.1975.10666649.
Cavalleri, A., D. Djurić, U. Maugeri, D. Branković, E. Visconti, and I. Rezman. 'Endocrinological Findings in Young Workers Exposed to Carbon Disulphide. I. Urinary Excretion of Total 17-Ketosteroids'. *La Medicina Del Lavoro* 57, no. 10 (1966): 566–72.
Cirla, A. M., P. A. Bertazzi, M. Tomasini, A. Villa, C. Graziano, R. Invernizzi, and R. Gilioli. 'Study of Endocrinological Functions and Sexual Behaviour in Carbon Disulphide Workers'. *La Medicina Del Lavoro* 69, no. 2 (1978): 118–29.
Cirla, and graziano. 'Health Impairment in Viscose-Rayon Workers with Carbon Disulfide Risks below 30 Mg/M3'. *G. Ital. Med. Lav.*, 3 (1981): 69–73.
El-Sobkey, M. K., A. A. Massoud, A. H. Abdel-Karim, and R. Fares. 'Serum Thyroxine, Serum Cholesterol and Its Fractions in Workers Exposed to Carbon Disulphide'. *The Journal of the Egyptian Public Health Association* 54, no. 5–6 (1979): 431–42.
Guo, Yinsheng, Yue Ma, Guoyuan Chen, and Jinquan Cheng. 'The Effects of Occupational Exposure of Carbon Disulfide on Sexual Hormones and Semen Quality of Male Workers From a Chemical Fiber Factory'. *Journal of Occupational and Environmental Medicine* 58, no. 8 (2016): e294-300. https://doi.org/10.1097/JOM.0000000000000823.
Hemminki, K., and M. L. Niemi. 'Community Study of Spontaneous Abortions: Relation to Occupation and Air Pollution by Sulfur Dioxide, Hydrogen Sulfide, and Carbon Disulfide'.

International Archives of Occupational and Environmental Health 51, no. 1 (1982): 55–63. https://doi.org/10.1007/BF00378410.
Lancranjan, I. 'Alterations of Spermatic Liquid in Patients Chronically Poisoned by Carbon Disulphide'. *La Medicina Del Lavoro* 63, no. 1 (1972): 29–33.
Ma, Ji-Ying, Jia-Jia Ji, null Qing Ding, Wei-Dong Liu, Si-Quan Wang, null Ning Wang, and Guo-Yuan Chen. 'The Effects of Carbon Disulfide on Male Sexual Function and Semen Quality'. *Toxicology and Industrial Health* 26, no. 6 (2010): 375–82. https://doi.org/10.1177/0748233710369127.
Meyer, C. R. 'Semen Quality in Workers Exposed to Carbon Disulfide Compared to a Control Group from the Same Plant'. *Journal of Occupational Medicine.: Official Publication of the Industrial Medical Association* 23, no. 6 (1981): 435–39. https://doi.org/10.1097/00043764-198106000-00018.
Pieleszek, A. '[The effect of carbon disulphide on menopause, concentration of monoamines, gonadotropins, estrogens and androgens in women]'. *Annales Academiae Medicae Stetinensis* 43 (1997): 255–67.
Takebayashi, T., K. Omae, C. Ishizuka, T. Nomiyama, and H. Sakurai. 'Cross Sectional Observation of the Effects of Carbon Disulphide on the Nervous System, Endocrine System, and Subjective Symptoms in Rayon Manufacturing Workers'. *Occupational and Environmental Medicine* 55, no. 7 (1998): 473–79. https://doi.org/10.1136/oem.55.7.473.
Takebayashi, Toru, Yuji Nishiwaki, Tetsuo Nomiyama, Takamoto Uemura, Tsuneyuki Yamauchi, Shigeru Tanaka, Haruhiko Sakurai, Kazuyuki Omae, and Japanese Rayon Worker's Health Study Group. 'Lack of Relationship between Occupational Exposure to Carbon Disulfide and Endocrine Dysfunction: A Six-Year Cohort Study of the Japanese Rayon Workers'. *Journal of Occupational Health* 45, no. 2 (2003): 111–18. https://doi.org/10.1539/joh.45.111.
Vanhoorne M. 'Preliminary Study of Toxicity of Carbon Disulphide and Hydrogen Sulphide in the Belgian Viscose Industry.' *G. Ital. Med. Lav*, 1981. 3, 57-68
Vanhoorne, M., F. Comhaire, and D. De Bacquer. 'Epidemiological Study of the Effects of Carbon Disulfide on Male Sexuality and Reproduction'. *Archives of Environmental Health* 49, no. 4 (1994): 273–78. https://doi.org/10.1080/00039896.1994.9937479.
Vanhoorne, M., A. Vermeulen, and D. De Bacquer. 'Epidemiological Study of Endocrinological Effects of Carbon Disulfide'. *Archives of Environmental Health* 48, no. 5 (1993): 370–75. https://doi.org/10.1080/00039896.1993.9936730.
Wägar, G., M. Tolonen, P. Tanner, and E. Helpiö. 'Serum Gonadotropins and Testosterone in Men Occupationally Exposed to Carbon Disulfide'. *Journal of Toxicology and Environmental Health* 11, no. 4–6 (1983): 691–701. https://doi.org/10.1080/15287398309530377.
Wink, A. 'Toxic Substances and Adrenocortical Hormones: Study on the Impact of Toxic Substances on the Excretion of Steroids in Humans.' MD Thesis, Wolters Noordhof NV Groningen, The Netherlands, n.d.
Zhou, S. Y., Y. X. Liang, Z. Q. Chen, and Y. L. Wang. 'Effects of Occupational Exposure to Low-Level Carbon Disulfide (CS2) on Menstruation and Pregnancy'. *Industrial Health* 26, no. 4 (1988): 203–14. https://doi.org/10.2486/indhealth.26.203.

Table S4: Parameters assembled into line of evidence and provided sufficient evidence.

Reference	Grouping	Lines of evidence	Species	Exposure weeks	Route of exposure	Effect dose (mg/kg/day)[1]	Observed effects (positive and negative)	Assessment of each line of evidence	Assessment of the integrated line of evidence	Modality[2]
In vitro mechanistic	*In vitro* mechanistic	ToxCast thyroid assay	ToxCast thyroid assay	/	/	/	Positive in "NCCT_QuantiLum_inhib_2_dn" assay and "NCCT_TPO_AUR_dn" assay investigating TPO activity with thiourea, one of the metabolite of carbon disulfide	Supporting evidence	Overall positive evidence for endocrine activity (T4 level)	T
Unpublished report, 2019	*In vivo* mechanistic	T3 and/or T4 levels	Rats	*	Oral	120 mg/kg	Significant decrease in T4 levels in F0 males and females and adult F1 male adults	Sufficient evidence.		T
Van Stee et al., 1986	*In vivo* mechanistic	T3 and/or T4 levels	Rabbits	12	Inhalation	948 mg/m3	Decreased T4 levels			T
Huang et al., 2012	EATS-mediated	Sperm morphology	Rats	10	Inhalation	1250 mg/m^3	Significant dose-related increased in abnormal sperm (teratospermia, increased number of sperm with folded tail, amorphous heads or without hooks	Sufficient changes observed by different route of exposure (non-standard and standard study designs)	Overall, positive evidence for adversity (sperm effect). Inconclusive results in Human.	E, A, S
Kumar et al., 1999	EATS-mediated	Sperm morphology	Rats	8	Intraperitoneal injections	200 mg/kg	Increased sperm-head shape abnormalities			E, A, S
Unpublished report, 2019	EATS-mediated	Sperm morphology	Rats	10*	Oral	120 mg/kg	In the F1-generation, significant increase in sperm cells with detached heads (4-fold of controls), at PND89-95			E, A, S
Huang et al., 2012	EATS-mediated	Sperm numbers	Rats	10	Inhalation	250 mg/m^3	Dose-related statistically significant decrease in sperm concentration			E, A, S
Kumar et al., 1999	EATS-mediated	sperm numbers	Rats	8	Intraperitoneal injections	100 mg/kg	Significant decreased in sperm count in cauda epididymis			E, A, S
Patel et al., 1999	EATS-mediated	Sperm numbers	Rats	4	Intraperitoneal injections	100 mg/kg	Fewer/absent sperm in the lumen (no information on statistics, incidence, severity). No effect in epididymal tissue			E, A, S
Tepe and Zenick, 1984	EATS-mediated	Sperm numbers	Rats	10	Inhalation	1896 mg/m^3	Significant decrease in epididymal and ejaculated sperm count			E, A, S
Unpublished report, 2019	EATS-mediated	Sperm numbers	Rats	10*	Oral	120 mg/kg	No effect in sperm count			E, A, S

Zenick et al., 1984	EATS-mediated	Sperm numbers	Rats	10	Inhalation	1896 mg/m^3	Significant decrease in semen sperm count at 7 and 10 weeks (-28% at week 7 and -36% at week 10 compared to baseline level)	Supporting evidence for adversity. Sufficient change in non-standard studies. No effect in the EOGRTS study (Unpublished report, 2019) may be related to differences in dose levels.		E, A, S
Unpublished report, 1983a	Parameters sensitive to but not diagnostic of EATS	Brain weight	Rats	13	Inhalation	158 mg/m^3	Significant dose-related decrease in absolute weight	Overall, sufficient evidence obsered in tow species and two route of exposure (oral and inhalation)	Positive evidence for brain findings, landing footsplay, follicle number and time to mating	N
Unpublished report, 1983b	Parameters sensitive to but not diagnostic of EATS	Brain weight	Rats	13	Inhalation	158 mg/m^3	Significant decrease in absolute weight			N
Unpublished report, 1983c	Parameters sensitive to but not diagnostic of EATS	Brain weight	Mice	13	Inhalation	950 mg/m^3	Significant decrease in absolute weight			N
Unpublished report, 2019	Sensitive to but not diagnostic of EATS	Brain weight	Rats	10*	oral	120 mg/kg	F0-generation : significant decrease in absolute brain weight (-6% and -5% relative to controls in males and females respectively) F1-generation: significant decrease in absolute brain weight (-5-10%), F2 generation: significant decrease in absolute brain weight in males (-5% at PND 21-23)			N

Unpublished report, 1983a	Sensitive to but not diagnostic of EATS	Brain histopathology	Rats	13	Inhalation	2528 mg/m^3	Axonal swelling of nerve fibers of the ventral and lateral funiculi of the spinal cord, segmental degeneration of fibers in the sciatic nerve in few animals		N
Unpublished report, 1983b	Sensitive to but not diagnostic of EATS	Brain histopathology	Rats	13	Inhalation	2528 mg/m^3	Axonal swelling of nerve fibers of the ventral and lateral funiculi of the spinal cord, with most commonly affected the thoracic cord, followed by lumbar and cervical cords. Segmental degeneration of fibers in the sciatic or tibial nerve in some animals (6/10 per sex)	Overall, sufficient evidence	N
Unpublished report, 1983c	Sensitive to but not diagnostic of EATS	Brain histopathology	Mice	13	Inhalation	2528 mg/m^3	Axonal degeneration and swelling in neurons		N
Unpublished report, 2019	Sensitive to but not diagnostic of EATS	Brain histopathology	Rats	10	Oral	120 mg/kg	No effect		N
Unpublished report, 2019	Sensitive to but not diagnostic of EATS	Brain morphometric	Rats	10*	Oral	120 mg/kg	Significant increase in the mean caudate putamen (striatum) width was noted in females at PND 21-22 or 76-90 . Significant decrease in the thickness of the corpus callosum, the bundle of nerve fibers that connect the two hemispheres, was also observed in males	Overall, sufficient evidence	N
Saillanfait et al., 1989	Sensitive to but not diagnostic of EATS	Presence of anomalies	Rats	Gestation Day 6-20	Inhalation	630 mg/m^3	Increased skeletal malformations (club foot) at maternal toxic dose	Overall, sufficient evidence.	N
Unpublished report, 1991	Sensitive to but not diagnostic of EATS	Presence of anomalies	Rabbits	Gestation Day 6-18	Inhalation	3792 mg/m^3	Increased external malformation (hydrocephaly) and skeletal malformation in presence of maternal toxicity		N
Saillanfait et al., 1989	Sensitive to but not diagnostic of EATS	Embryonic or fetal deaths and viable foetuses	Rats	Gestation Day 6-20	Inhalation	2528 mg/m^3	No effect		N

Unpublished report, 1991	Sensitive to but not diagnostic of EATS	Embryonic or fetal deaths and viable foetuses	Rabbits	Gestation Day 6-18	Inhalation	3792 mg/m3	Increased post-implantation losses, reduced number of live foetuses, no maternotoxicity at 1896 mg/m3	Overall, sufficient evidence. Absence of effect in rat in Saillanfait et al. compare to EOGRTS study (unpublished report, 2019) may be due to differences in study design	N
Unpublished report, 1992	Sensitive to but not diagnostic of EATS	Embryonic or fetal deaths and viable foetuses	Rats	14-day premating, mating, gestation up to PND 21	Inhalation	1554 mg/m3	Increased pup mortality, viaility and live litter size (in presence of maternal toxicity)		
Unpublished report, 2019	Sensitive to but not diagnostic of EATS	Embryonic or fetal deaths and viable foetuses	Rats	10*	oral	120 mg/kg	Increased post-implantation losses in both generation. Stronger effects in the F1 generation compare to F0 generation		N
Unpublished report, 2019	Sensitive to but not diagnostic of EATS	Functional observational battery	Rats	10*	oral	120 mg/kg	Decreased landing footsplay, not statistically significant but dose related in both males and females and above 20% difference compared to control	Overall, sufficient evidence.	N
Unpublished report, 2019	Sensitive to but not diagnostic of EATS	Number of ovarian follicles	Rats	10	Oral	120 mg/kg	Statistically significant decrease in primary and primordial follicle count (34 vs 49 in control), above historical control data (cohort F1A). Concomittant non statistically significant decrease in corporea lutea.	Overall, sufficient evidence.	N
Tepe and Zenick, 1984	Sensitive to but not diagnostic of EATS	Time to mating	Rats	10	Inhalation	1896 mg/m^3	Statistically significant decreased in ejaculation latencies after 4-week exposure and mount latency after 7-week exposure	Overall, sufficient evidence	N
Zenick et al., 1984	Sensitive to but not diagnostic of EATS	Time to mating	Rats	10	Inhalation	1896 mg/m^3	Decreased latency to ejaculation. No effects on mounts or intromissions		N
Unpublished report, 2019	Sensitive to but not diagnostic of EATS	Time to mating	Rats	10*	Inhalation	120 mg/kg	No effect on precoital time		N
Wronska-Nofer, 1980	Non-EATS mediated	Clinical chemistry: lipid	Rats	60	Inhalation	1000 mg/m^3	Increased total cholesterol and esterified. Effect seen after 15 month but not following 6-month		/

Unpublished report, 2019	Non-EATS mediated	Clinical chemistry: lipid	Rats	10*	Inhalation	120 mg/kg	No effect on cholesterol	Sufficient evidence following chronic exposure. Not investigated in the 90-day guideline studies. Not observed in EOGRTS.	
Lewis et al., 1999	Non-EATS mediated	coronary histopathology	Mice	20	Inhalation	1580 mg/m3	Increased arterial fatty deposit	Sufficient evidence; effect enhanced by fatty diet	/
Unpublished report, 1983c	Evidence of general toxicity	Survival	Mice	13	Inhalation	2528 mg/m^3	4/10 mice were found dead during the study	Sufficient evidence, in mice, high dose only	/
Tepe and Zenick, 1984	Evidence of general toxicity	Body weight	Rats	10	Inhalation	1896 mg/m^3	No effect on body weight in sexually active rats	Moderate changes in mice and rats, high dose only, around 10 % compare to control	/
Unpublished report, 1983a	Evidence of general toxicity	Body weight	Rats	13	Inhalation	2528 mg/m^3	Body weight decrease in males		/
Unpublished report, 1983b	Evidence of general toxicity	Body weight	Rats	13	Inhalation	2528 mg/m^3	Body weight decrease in males		/
Unpublished report, 1983c	Evidence of general toxicity	Body weight	Mice	13	Inhalation	2528 mg/m^3	Statistically significant treatment-related decrease in body weight gain male and female		/
Unpublished report, 1992	Evidence of general toxicity	Body weight	Rats	14-day premating, mating, gestation up to PND 21	Inhalation	1554 mg/m^3	Mean body weight and body weight gain significantly reduced througout gestation (4.5 to 10% changes compare to control). No effect during lactating period		
Unpublished report, 2019	Evidence of general toxicity	Body weight	Rats	10*	Oral	120 mg/kg	Decrease body weight gain in males and females P0 and F1, by about 10%		/
Zenick et al., 1984	Evidence of general toxicity	Body weight	Rats	10	Inhalation	1896 mg/m^3	Decreased body weight, around 10% compare to controls		/

Unpublished report, 1983a	Evidence of general toxicity	Haematology	Rats	13	Inhalation	2528 mg/m^3	Erythrocyte count depression, changes in platelet, eosinophils, lymphocytes	Sufficient evidence in both mice and rats, high dose only	/
Unpublished report, 1983b	Evidence of general toxicity	Haematology	Rats	13	Inhalation	2529 mg/m^3	Changes in leucocyte count in males, decreased haemoglobin and hematocrit in females		/
Unpublished report, 1983c	Evidence of general toxicity	Haematology	mice	13	Inhalation	2530 mg/m^3	Red blood cells, haemoglobin and haematocrit were significantly reduced in both sexes		/
Unpublished report, 2019	Evidence of general toxicity	Haematology	Rats	10*	Oral	120 mg/kg	Increased reticulocyte count		/
Unpublished report, 1983b	Evidence of general toxicity	Spleen weight	Rats	13	Inhalation	2528 mg/m^3	Increased weight in both sexes	Sufficient evidence in rats, high dose only	/
Unpublished report, 1983a	Evidence of general toxicity	Spleen weight	Rats	13	Inhalation	2528 mg/m^3	Increased weight in both sexes		/
Unpublished report, 2019	Evidence of general toxicity	Spleen weight	Rats	10*	Oral	120 mg/kg	Statiscally significant increase in relative weight, in P0 and F1, both sexes		/
Unpublished report, 1983b	Evidence of general toxicity	Spleen histopathology	Rats	13	Inhalation	2528 mg/m^3	Hemosiderin deposit in males and females	Sufficient evidence in rats and mice, high dose only	/
Unpublished report, 1983c	Evidence of general toxicity	Spleen histopathology	Mice	13	Inhalation	2528 mg/m^3	Hemosiderin deposit in all males and females		/
Unpublished report, 2019	Evidence of general toxicity	Spleen histopathology	Rats	10*	Oral	120 mg/kg	Increase incidence and severity of hemosiderine pigmentation		/
Unpublished report, 1983c	Evidence of general toxicity	Kidney histopathology	Mice	13	Inhalation	2528 mg/m^3	Nephropathy in 8/10 male rats, tubular syncytia in 9/10 males and 6/10 females	Sufficient evidence in mice	/
Unpublished report, 2019	Evidence of general toxicity	Retinal atrophy	Rats	10*	Oral	120 mg/kg	Increased incidence of retinal atrophy of the outer layer in males and females graded moderate in males and severe in females (P0 and F1)	Sufficient evidence in rats, severe effect in females	/
Unpublished report, 1983a	Evidence of general toxicity	Thymus weight	Rats	13	Inhalation	2528 mg/m^3	No effect		/

Unpublished report, 1983b	Evidence of general toxicity	Thymus weight	Rats	13	Inhalation	2528 mg/m^3	No effect	Sufficient evidence by oral route, not observed following inhalation exposure	/
Unpublished report, 1983c	Evidence of general toxicity	Thymus weight	Mice	13	Inhalation	2528 mg/m^3	No effect		/
Unpublished report, 2019	Evidence of general toxicity	Thymus weight	Rats	10*	Oral	120 mg/kg	Statistically significant dose-related decrease, in both sexes, P0 and F1		/
Unpublished report, 2019	Evidence of general toxicity	Thymus histopathology	Rats	10*	Oral	120 mg/kg	Increase incidence of lymphoid depletion, P0 and F1, graded minimal to slight	Sufficient evidence by oral route, not observed following inhalation exposure	/
Unpublished report, 1983a	Evidence of general toxicity	Thymus histopathology	Rats	13	Inhalation	2528 mg/m^3	No effect		/
Unpublished report, 1983b	Evidence of general toxicity	Thymus histopathology	Rats	13	Inhalation	2528 mg/m^3	No effect		/
Unpublished report, 1983c	Evidence of general toxicity	Thymus histopathology	Mice	13	Inhalation	2528 mg/m^3	No effect		/

[1] Dose level converted based on 1 ppm = 3,16 mg/m^3

[2] Modalities: Estrogen (E), Androgen (A), Steroidogenesis (S), thyroid (T) or not assignable to a specific modality (N)

* F0 male exposure: 10 weeks before mating, throughout mating until their termination; F0 females: 10 weeks before mating, throughout mating, gestation and at least 21 days after delivery up to the day before scheduled necropsy. F1 animals: dosed up to and including the day before scheduled necropsy.

*T4 measurements: at 11 weeks in males and 16 weeks females in F0-generation; at PND4, PND22 and PND 89 onward in F1 generation and at PND 4, 22 in F2-generation
